# Novel Lanthanide Complexes Synthesized from 3-Dimethylamino Benzoic Acid and 5,5′-Dimethyl-2,2′ Bipyridine Ligand: Crystal Structure, Thermodynamics, and Fluorescence Properties

**DOI:** 10.3390/molecules28248156

**Published:** 2023-12-18

**Authors:** Ya-Fan Hao, Su-Ling Xu, Quan Shi, Jin-Jin Zhao, Ning Ren, Jie Gao, Jian-Jun Zhang

**Affiliations:** 1 Testing and Analysis Center, College of Chemistry and Materials Science, Hebei Normal University, Shijiazhuang 050024, China; haoyafan1999@163.com (Y.-F.H.);; 2 Hebei Special Equipment Supervision and Inspection Institute, Shijiazhuang 050000, China; 3 Thermochemistry Laboratory, Dalian Institute of Chemical Physics, Chinese Academy of Sciences, Dalian 116023, China; shiquan@dicp.ac.cn; 4 Hebei Key Laboratory of Heterocyclic Compounds, College of Chemical Engineering & Material, Handan University, Handan 056005, China; ningren9@163.com; 5 Graduate School, Hebei GEO University, Shijiazhuang 050031, China

**Keywords:** supramolecular, lanthanide complexes, low-temperature heat capacity

## Abstract

Two isostructural lanthanide complexes were synthesized by solvent evaporation with 3-dimethylaminobenzoic acid and 5,5′-dimethyl-2,2′-bipyridine as ligands. The general formula of the structure is a [Ln(3-N,N-DMBA)_3_(5,5′-DM-2,2′-bipy)]_2_·2(3-N,N-DMHBA), Ln = (Gd(**1**), Tb(**2**)), 3-N,N-DMBA = 3-Dimethylamino benzoate, 5,5′-DM-2,2′-bipy = 5,5′-dimethyl-2,2′ bipyridine. Both complexes exhibited dimeric structures based on X-ray diffraction analysis. At the same time, infrared spectroscopy and Raman spectroscopy were used to measure the spectra of the complex. A thermogravimetric infrared spectroscopy experiment was performed to investigate the thermal stability and decomposition mechanism of the complexes. Measurements of the low-temperature heat capacity of the complexes were obtained within the temperature range of 1.9 to 300 K. The thermodynamic function was calculated by heat capacity fitting. In addition, the fluorescence spectra of complex **2** were studied and the fluorescence lifetime values were determined, and the energy transfer mechanism of complex **2** was elucidated.

## 1. Introduction

Lanthanide complexes have a wide range of applications in many fields due to their structural composition and ionic properties [1,2]. The inefficient light absorption of lanthanide ions is due to their spin-forbidden f-f electron leaps. The sensitization of lanthanide luminescence by efficient energy transfer through organic ligands was reported for the first time [3]. After this, lanthanide photoluminescent complexes began to be extensively studied. For example, lanthanide complexes can be used as luminescent materials and fluorescent probes. In recent years, how to effectively transfer energy to lanthanide ions and sensitize theirluminescence has become a hot topic for researchers. Therefore, the selection of organic ligands plays a crucial role in the synthesis of complexes with special structures and functions. Among organic ligands, aromatic carboxylic acid ligands are the most commonly used, because they have a strong oxygen supply capacity and help to form stable complexes with lanthanide ions. In addition, aromatic carboxylic acid ligands are also good chromophores, which help to absorb and transfer light energy and enhance the luminescence performance of lanthanide ions [4,5]. The oxygen in the carboxyl groups tend to form intermolecular hydrogen bonds, thus creating a supramolecular structure. Nitrogen-containing ligands as auxiliary ligands allow the complexes to form more novel structures. Nitrogen-containing heterocyclic ligands tend to enable higher fluorescence efficiency of the complexes.

Moreover, nitrogen-containing heterocyclic ligands are rigid ligands. The coordination site is fixed and cannot be rotated, which makes the structural framework of the complexes stable [6]. However, the poor thermal stability of complexes often limits their application in the field of luminescence. Therefore, we believe that it is very necessary to study them in terms of thermal analysis. Since the stability of the complexes is often closely related to the central metal ions and ligands, as well as isomerization and spatial effects, the stability of the complexes can be investigated by thermogravimetric and infrared techniques [7,8]. The obtained thermal analysis data can help us to clarify the reaction mechanism, so as to understand the nature of the ligand and the effect of the change in the complex structure on the material properties, which may guide the synthesis of some special complexes based on certain thermal decomposition mechanisms [9,10]. In addition, the thermodynamic properties for lanthanide complexes are of great value in theoretical studies and practical applications. For example, the molar heat capacity of a complex can be used to obtain the thermodynamic functions (enthalpy and entropy) of the complex. At the same time, the determination of the amount of molar heat capacity is very important for the study of chemical reaction processes and the improvement of thermodynamic theory [11,12,13].

This time, we still chose 5,5′-dimethyl-2,2′ bipyridine (5,5′-DM-2,2′-bipy) as the neutral ligand to improve the stability of the complex [14]. And new lanthanide complexes were synthesized using the new acidic ligand 3-dimethylamino benzoic acid (3-N,N-DMHBA). The effects of 5,5′-DM-2,2′-bipy and 3-N,N-DMHBA on lanthanide complexes were analyzed from the viewpoint of energy transfer. Two lanthanide complexes were obtained and characterized using single crystal method of X-ray diffraction, IR, Raman, elemental analysis, and XRD. The thermal stability of the complexes was studied using the TG-DSC/FTIR technique. The low-temperature heat capacities of the two complexes were also measured to analyze the possible chemical transformations at certain temperatures. The fluorescence properties and lifetime of complex **2** were studied. The luminescence of complex **2** was analyzed from the viewpoint of energy transfer.

## 2. Results and Discussion

### 2.1. Crystal Structure

We performed single-crystal diffraction of the two complexes using a Smart-1000 single-crystal diffractometer. The complexes were resolved using the intrinsic phase method of the SHElXT-2018 [15] structure solver for all structures. Optimization was performed using the SHELXL-2019 optimization software package. Coordinates and anisotropy were improved using the full-matrix least-squares method using OLEX2-1.5 software [16]. (CCDC numbers of the two complexes: CCDC 2268706(1); CCDC 2268708(2).)

**Figure 1:**
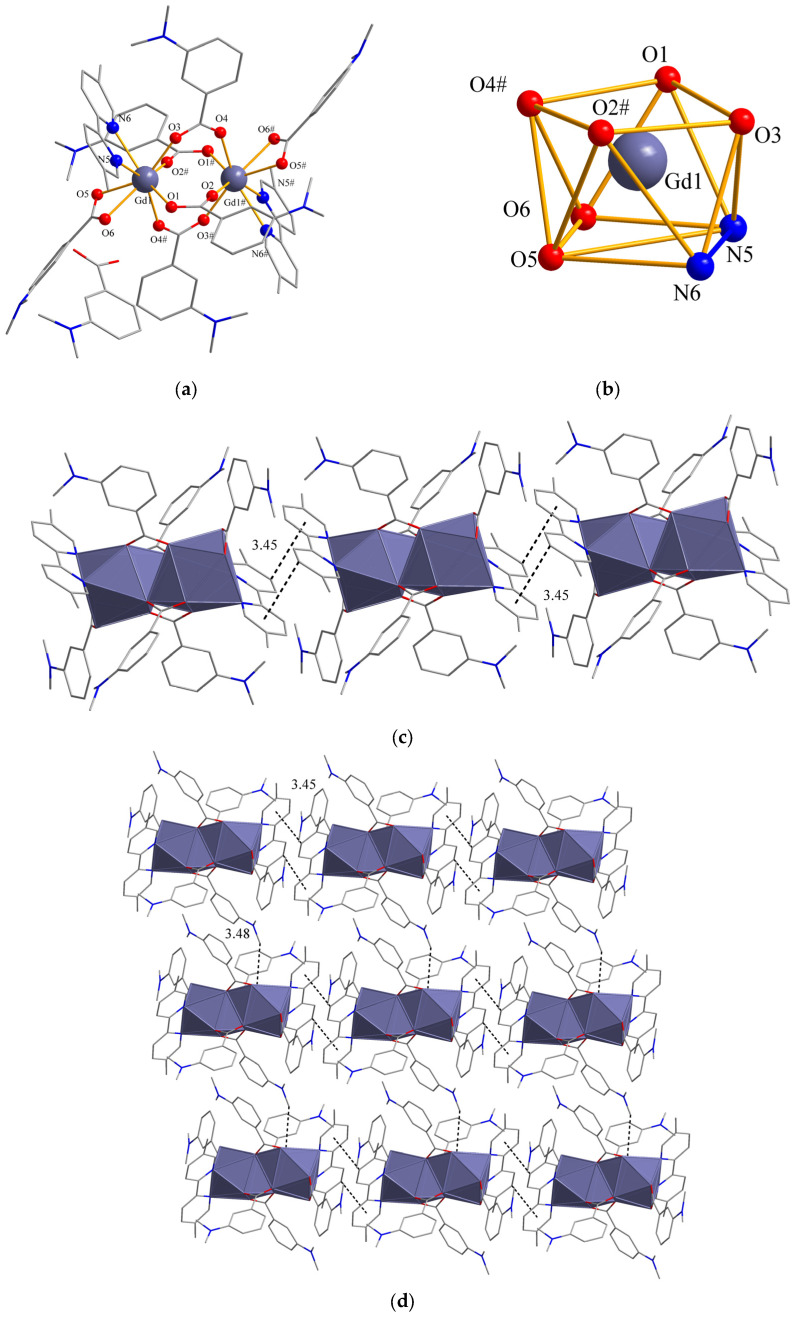
(**a**) Structural diagram of complex **1**, (**b**) conformation of complex **1**, (**c**) 1D chain view of complex **1** along the c-axis, (**d**) view of the laminate structure of complex **1** on the ab axis.

The single-crystal data derived from X-ray diffraction analysis show that complexes **1** and **2** are isostructural, crystallized in a monoclinic system, and *P2*_1_*/n* space groups (Table S1). The coordination number of complexes **1** and **2** is eight. The shape of complexes **1** and **2** was identified as a square antiprism by SHAPE software (v7.4). Next, we take Complex 1 as an example to study its crystal structure in detail. The structure of complex **1** is shown in Figure 1, where it can be seen that each structural unit is composed of two Gd^3+^ and six 3-N, N-DMBA, two neutral ligands, and two free 3-N, N-DMHBA (Figure 1a). The coordination environment of the two types of Gd^3+^ is the same, and both are 8-coordinated. Each Gd^3+^ ion is coordinated to six O atoms and two N atoms, with all O atoms coming from the 3-N, N-DMBA ligand, and two N atoms from the neutral ligands (Figure 1b). There are two coordination modes between Gd^3+^ and O atoms. One is a bridging bidentate (O1, O2^#^, O3, O4^#^), and the other is a bidentate (O5, O6). The bond lengths of Gd-O and Gd-N are shown in Table S2. The average bond length of Gd-O is 2.38 Å, and the average bond length of Gd-N is 2.60 Å. The average Gd-N distance in the binuclear complex is larger than the average Gd-O distance, which is consistent with the previously reported bond lengths [17]. This provides an important basis for the sequential order of loss of the two ligands in subsequent thermal decomposition analyses [18].

In the c-axis direction, the 5,5′-DM-2,2′-bipy of two neighboring structural units produces a C(44)-H(44)···πstacking interaction. So, a one-dimensional chain structure is formed between the two neighboring structural units with an interaction distance of 3.45 Å(C-H to the center of the pyridine ring), as shown in Figure 1c. In the a-axis direction, the dihedral angle of 3-N,N-DMBA between two neighboring structural units is 83.4°. This conformation brings the hydrogen atom (H9A) on C9 close enough to O3 to produce a C-H···O interaction [C(9)-H(9A)···O(3), 121.04°, 3.48 Å]. Two neighboring 3-N, N-DMBAs are linked together to form the final two-dimensional lamellar structure. Figure 1d shows that complex **1** forms a two-dimensional lamellar structure in the ac direction.

### 2.2. Spectroscopy Using Infrared and Raman Wavelengths

**Figure 2:**
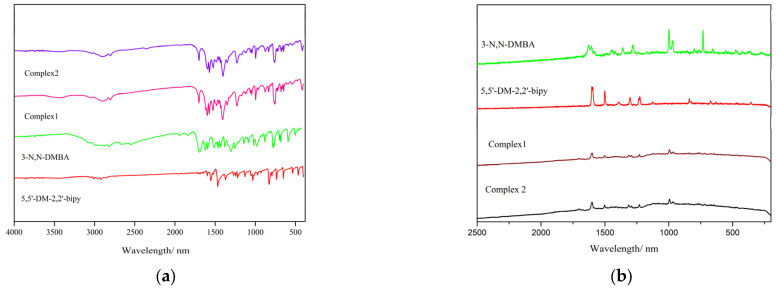
Infrared (**a**) and Raman (**b**) spectra of the complexes.

As shown in Figure 2a,b, the infrared and Raman spectra of the complexes and ligands were measured, and Table S3 shows the infrared and Raman wavelength data. It can be seen that the V_C=O_ of 3-N, N-DMBA disappears at 1676 cm^−1^(IR) and 1623 cm^−1^(R) after the formation of the complexes [19]. However, it is the heights of V_as(COO_^−^_)_ and V_s(COO_^−^_)_ that are supposed to be prominent that appear at 1533–1539 cm^−1^ (IR), 1600–1603 cm^−1^ (R), 1456 cm^−1^ (IR), and 1415 cm^−1^ (R), respectively. In addition, newly added Ln-O stretching vibration absorption peaks can also be observed at 419 cm^−1^ (IR) and 425–426 cm^−1^ (R). These changes can be considered as the coordination of Ln^3+^ ions and O atoms. For the neutral ligands, the wavelength of V_C=N_ change from 1554 cm^−1^ (IR) and 1510 cm^−1^ (R) to 1533–1539 cm^−1^ (IR) and 1509–1510 cm^−1^ (R), and an absorption peak can be seen at Ln-N at 266–269 cm^−1^, also appearing in the Raman spectrum. The above changes can be considered as the coordination of Ln^3+^ and N atoms [20].

### 2.3. XRD

**Figure 3:**
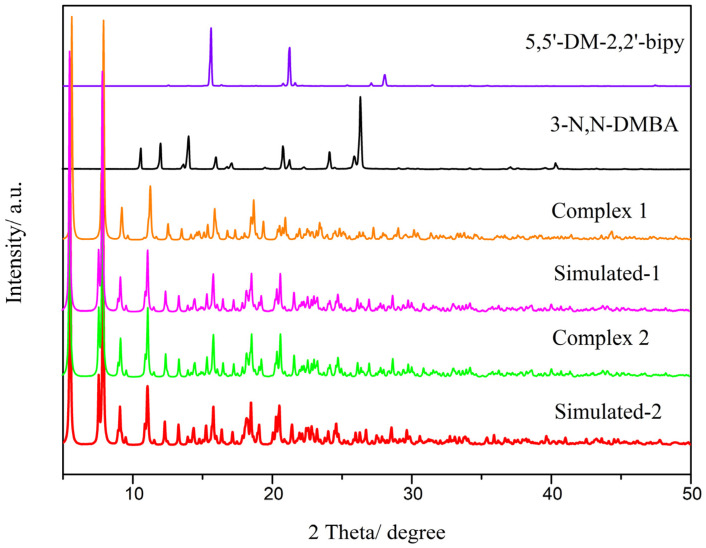
A comparison of the diffraction patterns of ligands and complexes observed using XRD.

A study of the ligands and complexes by X-ray powder diffraction was performed to illustrate their structures and phase purity. Firstly, in Figure 3, complexes **1** and **2** exhibit diffraction peaks different from those of the ligand in the diffraction pattern. This indicates that the [21,22] complex is not a mechanical mixture between two ligands, but rather, a coordination occurs to form a new compound. Secondly, a comparison was made between the simulated XRD calculated from the complex and the single crystal, and it was found that the positions of the diffraction peaks were basically the same. This provides evidence that the chemical composition of the sample is homogeneous and that there is only one crystalline phase.

### 2.4. Thermal Analysis

**Figure 4:**
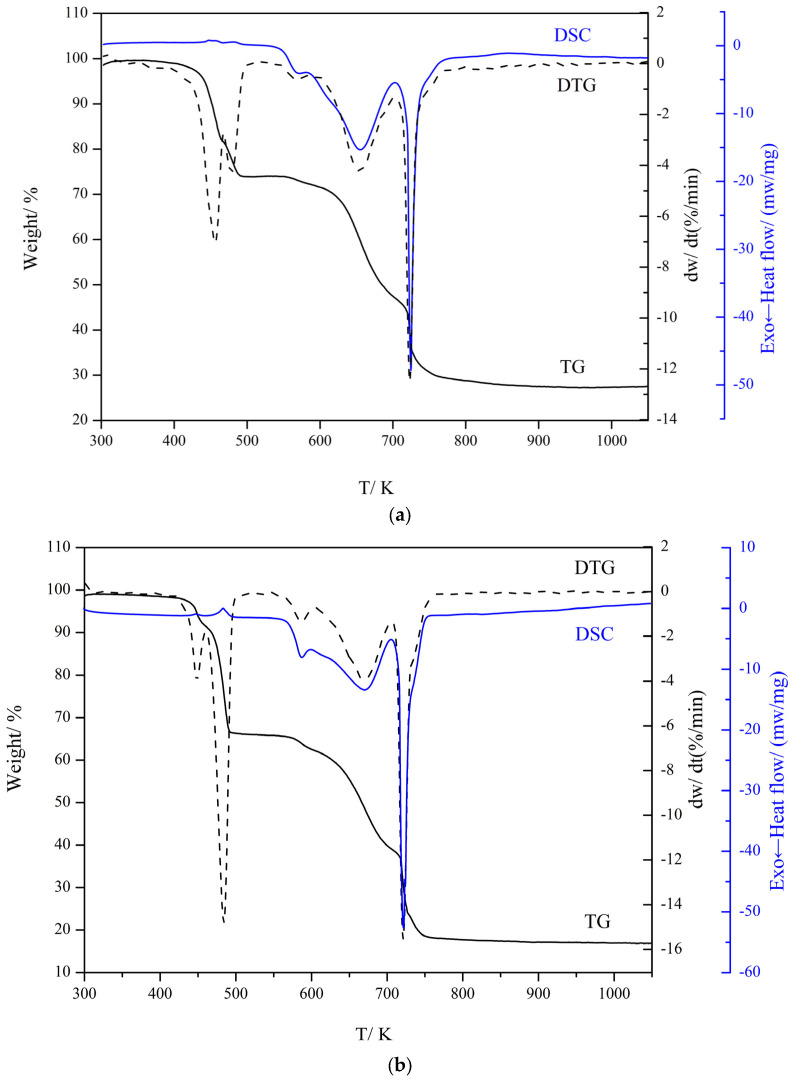
Diagrams of the TG-DTG/DSC curves for complexes **1** (**a**) and **2** (**b**).

Under dynamic air atmosphere simulation, the thermal stability and thermal decomposition mechanism of the composite were studied. Figure 4a,b show the TG-DTG/DSC images of complexes **1** and **2** at a heating rate of 10 K/min. The specific thermal analysis results are shown in Table 1.

The thermogravimetric curve of complex **1** has two steps. As shown in Figure 4a, the first step starts with the total loss of the two free 3-N,N-DMHBA at 445.05–560.95 K, as well as the partial loss of the neutral ligand. The mass loss is30.08%. The intermediate [Gd(3-N,N-DMBA)_3_(1–1/2x) (5,5′-DM-2,2′-bipy)]_2_ is formed. This weight loss step sequentially contains the two small fractions observable from the DTG. During the second step of weight loss at 560.95–762.25 K, the remaining neutral ligand and all 3-N,N-DMBA ligands involved in coordination are lost. The weightlessness of the second stepis 52.86%. The corresponding metal oxide Gd_2_O_3_is finally formed. The DTG curve in the temperature interval 560.95–762.25 K shows that it can be further divided into three small parts. During the entire thermal decomposition process, the weight loss is 82.94% (calculation result: 84.27%).

Figure 4b shows the TG-DTG/DSC curves of complex **2**. It is found that complex **2** is decomposed into twosteps. The first stepis the loss of two free 3-N, N-DMHBA, and partially coordinated neutral ligands at 478.15–533.15 K. The mass loss is 31.37%. [Tb(3-N,N-DMBA)_3_(1–1/2x)(5,5′-DM-2,2′-bipy)]_2_is formed. The first step of the decomposition can be further divided into two parts according to the DTG curve. The second weight loss step is in the temperature interval 574.55–730.05 K, where the remaining already coordinated neutral ligands and all the 3-N,N-DMBA are lost to form the final oxide (Tb_4_O_7_) with a weight loss of 49.50%. This weight loss step can be divided into three small parts according to the DTG curve. The total mass loss of complex **2** is 80.87% (Calcd: 81.72%).The above analysis shows that complexes **1** and **2** have good stability. We believe that the small differences in the weight loss portions of complex **1** and complex **2** are due to differences in the lanthanide ions.

### 2.5. Infrared Analysis of Fugitive Gases

**Figure 5:**
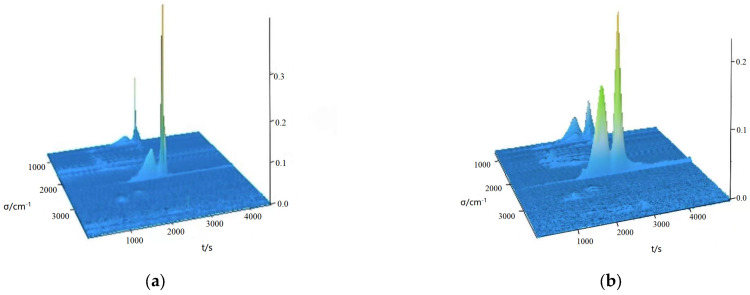
Three infrared stacking diagrams of the fugitive gas of complex **1** (**a**) and complex **2** (**b**).

**Figure 6:**
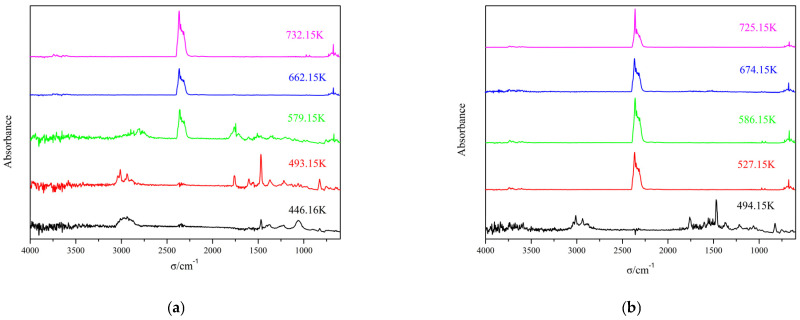
Two-dimensional IR spectrum of the fugitive gas of complex **1** (**a**) and complex **2** (**b**).

The three-dimensional spectra of the complexes were measured and plotted under simulated dynamic air conditions. The thermal decomposition mechanisms of the complexes were further investigated using the TG-DSC/FTIR technique. Figure 5a shows the 3D IR stacking of complex **1**, and Figure 5b shows the 3D IR stacking of complex **2**. Figure 6a,b shows the 2D analysis of complexes **1** and **2**, respectively.

A detailed analysis of complex **1** was carried out as an example. The 3D IR stacking map of complex **1** was studied. It was divided into two main steps. The first step was carried out at two temperatures of 446.16 K and 493.15 K, respectively, where water normally absorbs between 3762 and 3417 cm^−1^ [23]. In addition, there is a characteristic V_C-H_ band at 3014–2931 cm^−1^, a characteristic V_C=O_ band at 1754 cm^−1^, a characteristic V_C=C_ band at 1599–1558 cm^−1^, and a V_C-N_ absorption peak at 1373–1207 cm^−1^. This coincides with the decomposition of 3-N, N-DMHBA. At 493.15 K, distinct peaks in CO_2_ absorption can be seen at 2395–2229 cm^−1^ and 676 cm^−1^, respectively [24]. They are CO_2_ asymmetric stretching vibration and bending vibration, respectively. There are some organic small-molecule vibration peaks at 3756–3721 cm^−1^. For the second step of decomposition, three temperatures of 579.15 K, 662.16 K, and 732.15 K were selected based on the content of the decomposed material. Characteristic peaks of 2283–2395 cm^−1^ carbon dioxide can be observed at 579.15 K, 662.16 K, and 732.15 K [25]. This proves that the decomposition product carbon dioxide is produced at all three temperatures, mainly because of the breakdown of 3-N, N-DMBA. For complex **2**, the decomposition has similarities, and the characteristic peaks are similar to those of complex **1**, so we do not describe them too much.

### 2.6. Heat Capacity

In the presence of constant pressure (about 1.3 MPa), the molar heat capacities of the complexes were measured at 1.9 K to 300 K using a quantum design physical property measurement system (PPMS). The temperature interval below 100 K is logarithmic, and it is 10 K at 100–300 K. The temperature increase at each data point is 2%.

**Figure 7:**
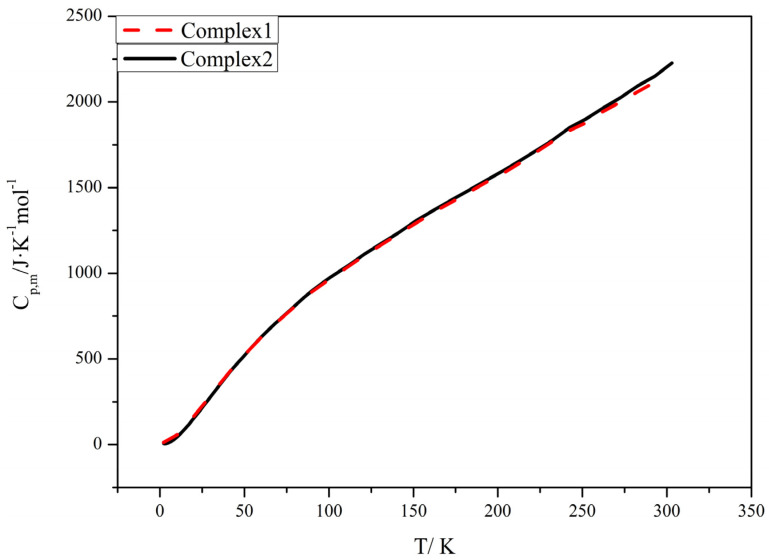
Heat capacity and temperature profiles of complexes **1** and **2**.

Figure 7 shows the corresponding heat capacity curves. Based on the figure, it is easy to see that the measurement range corresponds to a smooth curve. In this temperature range, the complexes are thermochemically stable.

No thermal anomalies, such as conjugation, phase change, and decomposition, were observed. Since the structures of complexes **1** and **2** are the same, the two heat capacity curves overlap highly. The enthalpy and entropy of a substance are important parameters in thermodynamics.In addition, their values are closely related to the molar heat capacity of the substance. Therefore, the values of the enthalpy and entropy of a substance can [26] be further deduced from the molar heat capacity of the substance. Using the least-squares method, a polynomial equation was obtained for complexes **1** and **2** by fitting the folded temperature x to experimentally determined molar heat capacity values [27] (x = [*T* − (*T*_max_ + *T*_min_)/2]/[(*T*_max_ − *T*_min_)/2]).

Complex **1**: C_p, m_/J·K^−1^·mol^−1^ = 1288.66375 + 883.58388x + (−240.90116)x^2^ + 243.91689x^3^ + 695.76904x^4^ + 70.64029x^5^ + (−1762.00968)x^6^ + (−197.89225)x^7^ + 1116.70353x^8^.

R^2^ = 0.99999; SD = 2.58687.

Complex **2**: C_p, m_/J·K^−1^·mol^−1^ = 1311.35943 + 899.76811x + (−265.69445)x^2^ + 134.93641x^3^ + 740.31286x^4^ + 494.24414x^5^ + (−1743.90122)x^6^ + (−415.26871)x^7^+ 1072.74812x^8^.

R^2^ = 0.99998; SD = 3.03888.

Table S4 shows the experimental molar heat capacities and the smoothed molar heat capacities of complexes **1** and **2**. Based on thermodynamics and polynomial equations, the thermodynamic functions of complexes **1** and **2** in the 3 K range were calculated with 298.15 K as the reference standard temperature. The thermodynamic functions are shown in Table S5.


$$H_{T}-H_{298.15}=\int_{298.15}^{T}Cp,mdT\;S_{T}-S_{298.15}=\int_{298.15}^{T}{Cp,mT}^{-1}dT$$

### 2.7. Solid-State Absorption Spectroscopy

**Figure 8:**
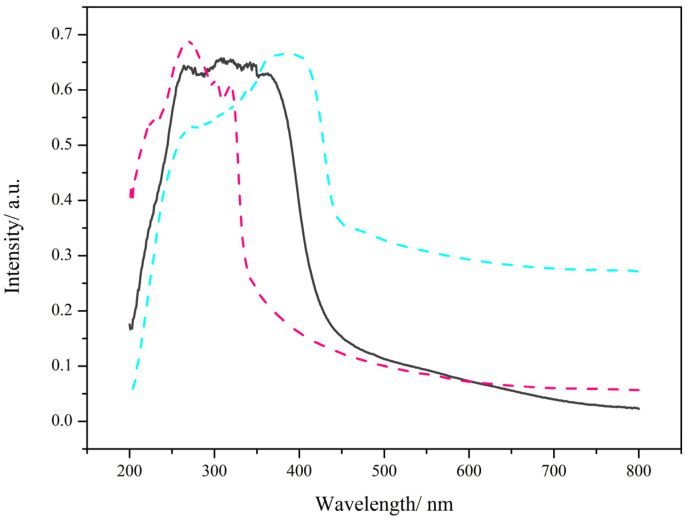
The black line is the absorption spectrum of complex **2**; the pink line is the absorption spectrum of 5,5′-DM-2,2′-bipy; the blue line is the absorption spectrum of 3-N,N-DMHBA.

The solid-state absorption spectrum for complex **2** displays an absorption band in the range of265 to 346 nm, which is attributed to the absorption of two ligands. The maximum absorption bands for 5,5′-DM-2,2′-bipy at 269, 301, and 320 nm are due to the π-π* transitions of the aromatic ring, and for 3-N,N-DMHBA at 270 and 387 nm are due to the n-π* transitions of the aromatic ring (Figure. 8). Though the broad absorption band of complex 2 shows a difference in shape with the solid-state UV absorption spectra of 5,5′-DM-2,2′-bipy and 3-N,N-DMHBA (Figure 8), the results of both ligands should be combined.

### 2.8. Fluorescent Properties

**Figure 9:**
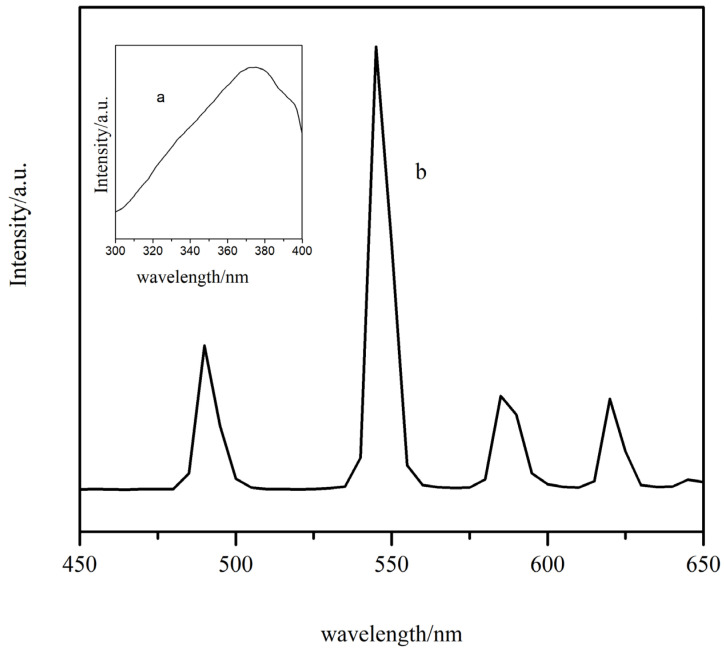
Fluorescence spectra of complex 2 ((**a**): excitation spectrum; (**b**): emission spectrum).

**Figure 10:**
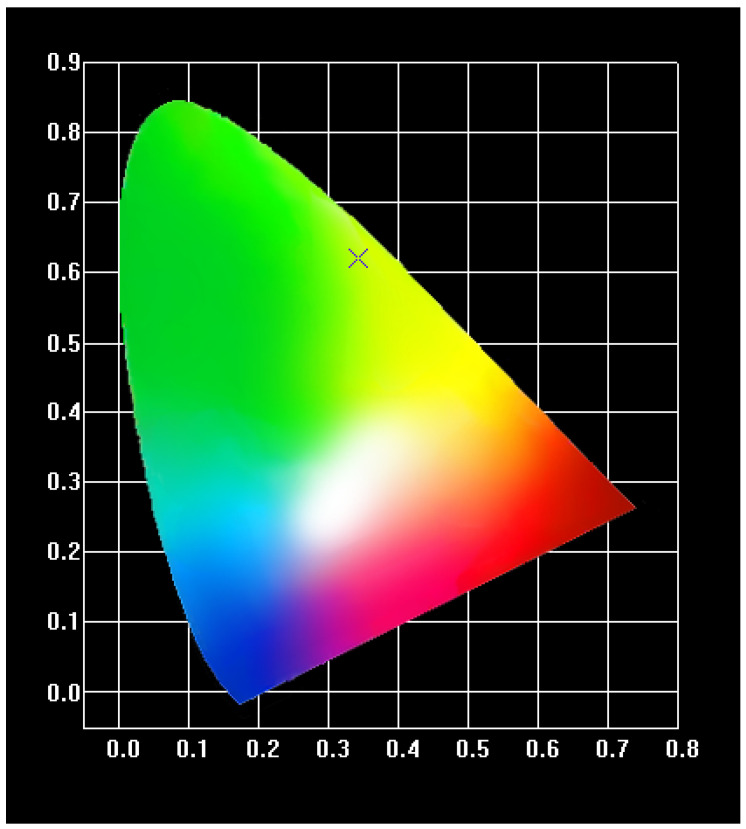
Color coordinates of complex **2**. (The × is the luminescent colors of complex **2**.).

**Figure 11:**
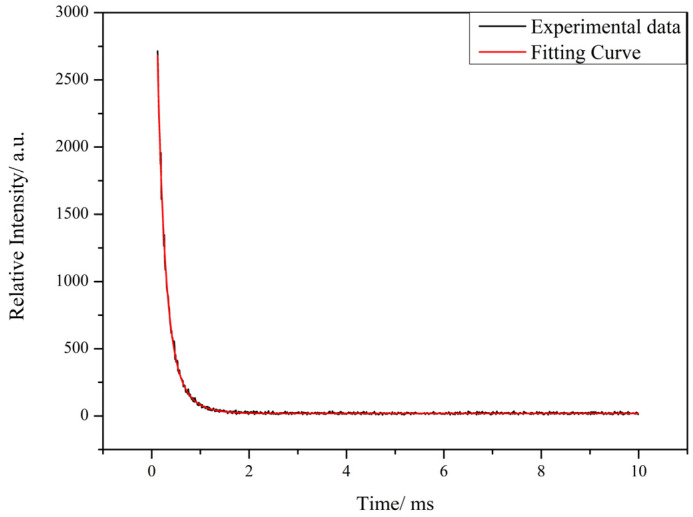
Fluorescence lifetime of complex **2**.

In Figure 9a,b, the excitation and emission spectra of complex **2** are shown, respectively. Figure 9a shows a strong absorption band between 300 and 400 nm. This is the result of the *π*→π* electron transition. The emission spectra of the complexes were measured at 365 nm. It can be seen that four characteristic emission bands of Tb^3+^ appear at 490 nm, 545 nm, 585 nm, and 620 nm. This is attributed to the deactivation of the ^5^D_4_ excited state of Tb^3+^ to the ^7^F_J_ (J = 6, 5, 4, 3) ground state. In addition, the jump peak of ^5^D_4_→^7^F_5_ at a wavelength of 545 nm is the most pronounced [28], which is the main reason for the green glow of the Tb^3+^ complexes. Then, we imported the emission spectrum of complex **2** into CIE software (v1.4.3-4) to obtain the green coordinates (0.3935, 0.6006) (Figure 10). Due to the strong characteristic luminescence of Tb^3+^, the fluorescence decay curve of complex **2** was measured at room temperature (Figure 11).

The fluorescence decays of complex **2** are biexponential decays. The results obtained indicate that Tb^3+^ as the luminescent center mainly occupies two lattice sites, and its fluorescence intensity and fluorescence lifetime follow the following equations: I(t) = B_1_exp(−t/τ_1_) + B_2_exp(−t/τ_2_); τ = (B_1_τ_1_^2^ + B_2_τ_2_^2^)/(B_1_τ_1_ + B_2_τ_2_) [29]. The τ_1_ and τ_2_ obtained from the fitted curves are 0.28 ms and 0.64 ms, and the ratios of τ_1_ and τ_2_ are 46.55% and 53.45%, respectively. The fluorescence lifetime of complex **2** was calculated to be 0.4709 ms.

**Figure 12:**
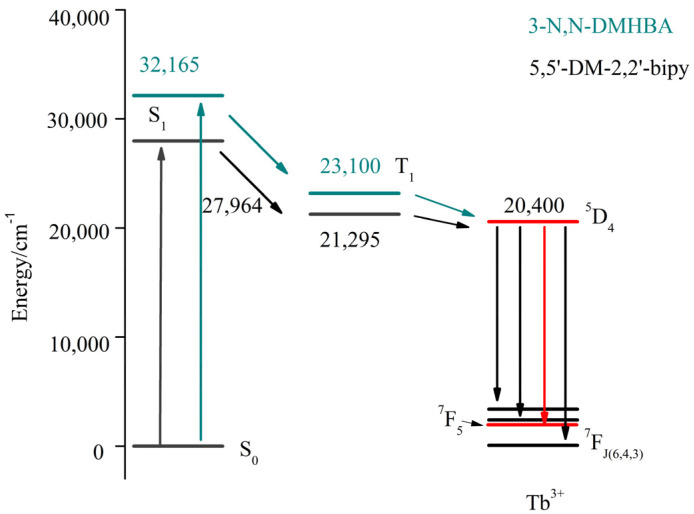
Energy transfer mechanism of complex **2**.

To explain the luminescence of lanthanide complexes, the mechanism of energy transfer from ligand to metal has been extensively investigated. Energy can be transferred efficiently from ligand to metal when the difference between the triplet and singlet states of the ligand is ≥5000 cm^−1^ and the lowest triplet state of the ligand is 2000–5000 cm^−1^ higher than the lowest emission energy level of the metal ion [30,31]. The geometric configurations were optimized at the B3LYP/6-311G level by the Gaussian 09 program using density functional theory (DFT). The singlet- and triplet-state energies of the ligand were also calculated. The excited singlet and lowest excited triplet states of 5,5′-DM-2,2′-bipy are 27,964 and 21,295 cm^−1^, respectively, and the excited singlet and lowest excited triplet states of 3-N,N-DMHBA are 32,165 and 23,100 cm^−1^, respectively. The lowest triplet and singlet energy levels of the ligand are greater than 5000 cm^−1^. After reviewing the literature [32], the lowest emission energy level of Tb^3+^ is 20,430 cm^−1^. Thus, the energy differences between the lowest triplet-state energy levels of 5,5’-DM-2,2’-bipy and 3-N,N-DMHBA and Tb^3+^are 865 and 2670 cm^−1^, respectively. The above data indicate that the introduction of acidic ligands can increase the fluorescence intensity of the complexes and effectively sensitize Tb^3+^. Figure 12 shows the energy transfer mechanism of complex **2**.

## 3. Experimental Part

### 3.1. Experimental Instruments

A Smart-1000 single-crystal diffractometer was used to perform X-ray diffraction on complexes **1** and **2**. Measurements were performed at room temperature using the Mo target Kα (λ = 0.71073 Å). The contents of C, H, and N were determined on a Vario-EL III elemental analyzer. The Fourier transform infrared spectrometer BRUKER TENSOR27 was used for infrared analysis. Raman spectra were recorded by a VERTEX-70 FTIR-RAMAN II (BRUKER, Mannheim, Germany) with 64 scans at 300 mW and liquid-nitrogen-cooled LnGaAs. X-ray powder diffraction was performed using a Bruker D8 with copper radiation (λ = 1.5418 Å). As a result of thermal decomposition, the complexes decomposed and the 3D infrared spectra of the fugitive gases were determined through combined use of the NETZSCH STA 449 F3 simultaneous analyzer and the BRUKER TENSOR27 Fourier transform infrared spectrometer at a heating rate of 10 K/min while simulating dynamic air. The molar heat capacities of the complexes were measured at 1.9 K–300 K using a quantum design physical property measurement system (PPMS) under constant pressure (about 1.3 mPa). The temperature intervals were logarithmic below 100 K and 10 K at 100–300 K, and the temperature increase for each data point was 2%. The solid UV spectra were tested using an Agilent Cary 60 UV-Vis spectrophotometer. The solid-state UV spectra of the complexes and ligands were measured under a xenon lamp light source (80 Hz). An Edinburgh integrated steady-state transient fluorescence spectrometer FS5 was used to measure the fluorescence lifetime and spectrum of complex **2** at room temperature and pressure.

### 3.2. Synthesis of Experimental Materials and Complexes

3-N,N-DMHBA, 5,5′-DM-2,2′-bipy, ethanol, and lanthanide nitrate are analytical pure reagents that do not require purification for use.

**Figure 13:**
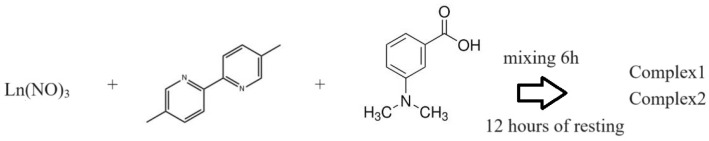
Synthesis process of the complexes.

We dissolved 0.06 mol of 3-N,N-DMHBA and 0.02 mol of 5,5′-DM-2, 2′-bipy in 7 mL ethanol, and adjusted the pH of the mixed ligand solution to 6.2–6.7 with 1 mol/L sodium hydroxide solution. We added another 0.02 mol of lanthanide ion nitrate, stirred for 7 h, and let the solution stand for 14 h. Then, we filtered, washed, and placed the filtrate in a 50 mL beaker, waiting for 7 days to obtain the complexes. The synthesis of complexes **1** and **2** is shown in Figure 13.

Elemental analysis: C_96_H_106_Gd_2_N_12_O_16_(%), Calcd: C: 57.70; N: 8.41; H: 5.35. Found: C: 57.59; N: 8.27; H: 5.42. C_96_H_106_Tb_2_N_12_O_16_(%), Calcd: C: 57.60; N: 8.40; H: 5.34. Found: C: 57.54; N: 8.23; H: 5.37.

## 4. Conclusions

Two new Lanthanide complexes [Ln(3-N,N-DMBA)_3_(5,5′-DM-2,2′-bipy)]_2_·2(3-N,N-DMBA), Ln = (Gd(**1**), Tb(**2**)), were synthesized. Complexes **1** and **2** were *P21/n* space point groups in a monoclinic crystal system. And they can be obtained through C-H···*π* and C-H···O intermolecular hydrogen bonds to form supramolecular one- and two-dimensional structures. The structures of the coordination geometry of complexes 1 and 2 were further studied by combining Raman spectroscopy, infrared spectroscopy, and XRD. The stability of the complexes was measured by the thermogravimetric infrared coupling method. It was found that the complexes started to decompose at about 445 and 478 K and eventually formed the corresponding lanthanide oxides with good stability.

In addition, the molar heat capacities of the complexes were measured from 1.9 K to 300 K. It was found that they increased with increasing temperature. And the heat capacity was fitted to an empirical model, as were the basic thermodynamic functions of the complexes, such as enthalpy and entropy. The new lanthanide complexes synthesized based on the above determination method can be developed as new low-temperature heat-resistant materials to some extent. By incorporating lanthanide’s emission spectrum into the color coordinate software, it can be seen that its coordinates are within the green light range. Its fluorescence lifetime was also measured to be up to 0.4709 ms. From the perspective of energy transfer, 3-N,N-DMHBA are the sensitizers of Tb^3+^.

## Figures and Tables

**Table 1:** Thethermal decomposition data for complexes **1** and **2**.

	Steps	Temperature Range/K	DTG T_p_/K	Mass Loss Rate/%		Probable Expelled Groups	Intermediate and Residue
				Found	Calcd		
1	I	445.05–560.95	490.8	30.08	34.97 ^a^	2(3-N,N-DMHBA)+x(5,5′-DM-2,2′-bipy)	[Gd(3-N,N-DMBA)_3_(1-1/2x)(5,5′-DM-2,2′-bipy)]_2_

	II	560.95–762.25	640.15	52.86		(2-x)(5,5′-DM-2,2′-bipy)	[Gd(3-N,N-DMBA)_3_]_2_
					49.29 ^b^	6(3-N,N-DMBA)	Gd_2_O_3_

				82.94	84.27 ^c^		
2	I	478.15–533.15	520.4	31.37	35.79 ^a^	2(3-N,N-DMHBA)+x(5,5′-DM-2,2′-bipy)	[Tb(3-N,N-DMBA)_3_(1-1/2x)(5,5′-DM-2,2′-bipy)]_2_

	II	574.55–730.05	673.25	49.50		(2-x)(5,5′-DM-2,2′-bipy)	[Tb(3-N,N-DMBA)_3_]_2_
					46.82 ^b^	6((3-N,N-DMBA)	1/2Tb_4_O_7_
	
				80.87	81.72 ^c^		

^a^: theoretical total mass loss of 2(3-N,N-DMHBA) and 2(5,5’-DM-2,2’-bipy); ^b^: theoretical total mass loss of 6(3-N,N-DMBA); ^c^: total loss rate.

## Data Availability

The data are available in this article and the Supplementary Materials.

## Supplementary Materials

The following supporting information can be downloaded at: https://www.mdpi.com/article/10.3390/molecules28248156/s1, Table S1. Crystallographic data for complexes **1** and **2**. Table S2. Selected Bond lengths [Å] for complexes **1** and **2**. Table S3. Infrared and Raman spectral data of complexes and ligands. Table S4. Experimental molar heat capacity of complexes **1** and **2** at a pressure of 1.3 mPa. Table S5. Thermodynamic function values of complex **1** and complex **2** at temperatures of 1.9–300 K.
