# Supplementary material for: Novel Lanthanide Complexes Synthesized from 3-Dimethylamino Benzoic Acid and 5,5′-Dimethyl-2,2′ Bipyridine Ligand: Crystal Structure, Thermodynamics, and Fluorescence Properties
Source: Molecules. 2023 Dec 18;28(24):8156. doi: 10.3390/molecules28248156 (PMC10745311; doi:10.3390/molecules28248156)
Supplement: Supplementary file 1 [file molecules-28-08156-s001.zip › molecules-2639698-supplementary.pdf]

Table S1. Crystallographic data for complexes **1** and **2.**

	Complex **1**	Complex **2**
Empirical formula	$C_{96}H_{106}Gd_2N_{12}O_{16}$	$C_{96}H_{106}N_{12}O_{16}Tb_2$
Formula weight	1998.42	2001.76
Temperature/K	293(2)	293(2)
Crystal system	monoclinic	monoclinic
Space group	$P2_1/n$	$P2_1/n$
a/Å	12.0529(12)	12.0882(11)
b/Å	31.067(3)	31.067(3)
c/Å	12.3932(13)	12.3989(12)
α/°	90	90
β/°	101.753(3)	101.837(4)
γ/°	90	90
Volume/Å^3	4543.3(8)	4557.3(7)
Z	2	2
ρ_{calc}g/cm^3	1.461	1.459
μ/mm^{-1}	1.520	1.612
F(000)	2044.0	2048.0
Crystal size/mm^3	0.48 × 0.22 × 0.03	0.3 × 0.15 × 0.07
Radiation	MoKα (λ = 0.71073)	MoKα (λ = 0.71073)
2Θ range for data collection/°	3.692 to 49.998	3.604 to 50.034
Index ranges	-11 ≤ h ≤ 14, -32 ≤ k ≤ 36, -14 ≤ l ≤ 14	-14 ≤ h ≤ 14, 0 ≤ k ≤ 36, 0 ≤ l ≤ 14
Reflections collected	22209	8002
Independent reflections	7981 [R_{int} = 0.1015, R_{sigma} = 0.0908]	8002 [R_{int} = ?, R_{sigma} = 0.1703]
Data/restraints/parameters	7981/6/579	8002/105/567
Goodness-of-fit on F^2	1.117	1.112
Final R indexes *[I>=2σ (I)]*	R_1 = 0.0773, wR_2 = 0.1755	R_1 = 0.1147, wR_2 = 0.2361
Final R indexes [all data]	R_1 = 0.1025, wR_2 = 0.1889	R_1 = 0.1896, wR_2 = 0.2706
Largest diff. peak/hole / e Å^{-3}	1.66/-2.83	1.97/-2.90

Table S2. Selected Bond lengths [Å] for complexes **1** and **2**.

Complex 1	Bond length/Å	Complex 2	Bond length/Å
Gd(1)-O(3)	2.313(5)	Tb(1)-O(3)	2.267(9)
Gd(1)-O(2)#1	2.316(6)	Tb(1)-O(2)#1	2.297(9)
Gd(1)-O (4)#1	2.344(6)	Tb(1)-O(1)	2.317(10)
Gd(1)-O(1)	2.363(6)	Tb(1)-O(4)#1	2.336(10)
Gd(1)-O(5)	2.460(6)	Tb(1)-O(5)	2.446(10)
Gd(1)-O(6)	2.486(6)	Tb(1)-O(6)	2.478(9)
Gd(1)-N(5)	2.599(7)	Tb(1)-N(5)	2.555(11)
Gd(1)-N(6)	2.603(7)	Tb(1)-N(6)	2.580(12)

Symmetry transformations used to generate equivalent atoms:1-x,2-y,-z

Table S3. Infrared and Raman spectral data of complexes and ligands.

**complexes**	$\nu_{C=N}$		δ_{C-H}		$\nu_{C=O}$		$\nu_{as(COO-)}$		$\nu_{s(COO-)}$		$\nu_{(Ln-O)}$		$\nu_{(Ln-N)}$
	**IR**	**R**	**IR**		**IR**	**R**	**IR**	**R**	**IR**	**R**	**IR**	**R**	**R**
3-N,N-DMHBA					1676	1623							
5,5'-DM-2,2'-bipy	1554	1597	827	736									
1	1533	1510	862	788			1533	1600	1456	1415	418	425	266
2	1539	1509	861	788			1539	1603	1456	1415	418	426	269

Table S4. Experimental molar heat capacity of complexes **1** and **2** at a pressure of 1.3 mPa.

**T/K**	Complex1		Complex2	
	$C_{p,m}(J\cdot K^{-1}\cdot mol^{-1})$		$C_{p,m}(J\cdot K^{-1}\cdot mol^{-1})$	
	**Exp.**	**smooth**	**Exp.**	**smooth**
1.936	1.736	1.744	6.546	6.567
2.034	1.741	1.722	6.431	6.385
2.146	1.717	1.723	6.140	6.153
2.267	1.734	1.737	5.840	5.833
2.396	1.797	1.795	5.550	5.568
2.532	1.880	1.876	5.374	5.357
2.675	1.984	1.988	5.206	5.218
2.826	2.159	2.165	5.137	5.132
2.988	2.408	2.394	5.118	5.114

3.159	2.666	2.676	5.158	5.161
3.339	3.027	3.027	5.301	5.303
3.535	3.472	3.466	5.557	5.555
3.737	3.989	3.999	5.913	5.907
3.951	4.642	4.623	6.384	6.403
4.177	5.376	5.404	7.048	7.029
4.417	6.353	6.334	7.762	7.755
4.672	7.406	7.390	8.599	8.627
4.938	8.590	8.635	9.704	9.678
5.222	10.143	10.113	10.888	10.895
5.524	11.777	11.769	12.261	12.270
5.843	13.549	13.587	13.848	13.829
6.177	15.604	15.537	15.572	15.582
6.530	17.645	17.709	17.569	17.554
6.907	20.156	20.140	19.856	19.911
7.310	22.868	22.842	22.627	22.572
7.729	25.701	25.723	25.434	25.435
8.171	28.876	28.856	28.474	28.481
8.640	32.349	32.370	31.932	31.950
9.136	36.303	36.293	35.915	35.900
9.661	40.677	40.682	40.321	40.342
10.223	45.527	45.503	45.230	45.165
10.814	50.845	50.918	50.483	50.594
11.440	56.904	56.803	56.682	56.607
12.097	63.127	63.185	63.128	63.114
12.791	70.118	70.126	69.993	70.011
13.523	77.804	77.766	77.616	77.652
14.304	86.016	86.038	86.130	86.085
15.127	95.033	95.074	95.243	95.289
15.997	104.88	104.809	105.191	105.137
16.914	115.107	115.070	115.675	115.650
17.883	126.093	126.241	127.035	127.171
18.913	138.652	138.595	139.904	139.849
20.001	151.874	151.814	153.323	153.208
21.149	165.515	165.501	166.968	167.038
22.363	180.032	180.167	181.858	181.907
23.644	196.042	195.876	198.194	198.158
25.001	212.585	212.713	215.611	215.629
26.441	230.632	230.585	234.164	234.162
27.961	249.666	249.658	253.697	253.638
29.563	269.724	269.701	274.081	274.137

31.262	290.938	291.143	295.879	295.885
33.057	313.710	313.292	319.005	319.053
34.953	336.044	336.367	343.302	343.076
36.957	360.817	360.806	368.061	368.400
39.087	387.136	387.145	395.220	395.068
41.331	414.230	414.077	422.759	422.629
43.707	441.221	441.400	450.431	450.773
46.218	469.015	468.547	479.499	478.795
48.867	496.421	497.004	507.237	508.000
51.668	527.710	527.708	539.127	538.824
54.633	560.357	559.931	571.746	571.820
57.771	591.865	592.037	605.207	605.089
61.080	624.706	624.927	638.757	638.818
64.583	659.350	659.191	673.022	673.131
68.290	693.698	693.486	707.916	707.536
72.210	727.508	727.485	742.078	742.254
76.353	763.007	763.653	778.585	778.848
80.722	802.692	802.010	818.468	818.467
85.361	841.526	842.342	859.691	859.606
90.269	882.432	881.255	900.226	900.044
95.462	918.807	917.380	939.031	936.723
100.935	957.782	961.699	977.190	981.604
111.003	1023.140	1022.350	1042.716	1041.559
121.057	1092.973	1090.920	1112.122	1110.862
131.163	1154.592	1156.108	1175.418	1175.152
141.264	1219.261	1218.385	1237.319	1239.319
151.366	1280.560	1281.395	1305.646	1303.710
161.468	1342.287	1341.364	1364.890	1365.424
171.567	1398.496	1398.579	1422.122	1422.042
181.678	1454.012	1455.235	1478.177	1478.390
191.768	1512.694	1510.297	1534.817	1534.561
201.874	1564.135	1566.248	1591.321	1591.360
211.984	1625.891	1625.425	1649.956	1650.445
222.114	1690.866	1691.053	1712.531	1711.206
232.241	1757.398	1758.339	1775.148	1778.933
242.377	1821.642	1817.217	1849.368	1843.908
252.463	1868.779	1874.819	1902.594	1906.413
262.563	1935.616	1930.864	1965.933	1963.852
272.659	1988.499	1991.568	2025.936	2028.426
282.784	2049.220	2044.053	2093.430	2089.213
292.868	2103.772	2113.671	2150.185	2155.543

303.008	2198.433	2194.344	2227.164	2225.188

When T = 1.9-20 K, the standard uncertainty u is u (p) = 0.10mPa, u (T) = 0.01 K, and the relative uncertainty is ur($C_{p, m}$) = 1.73%. When T = 20-300 K, the standard uncertainty u is u (p) = 0.10 mPa, u (T) = 0.02 K, and the relative uncertainty is ur ($C_{p, m}$) = 0.75%.

Table S5. Thermodynamic function values of complex **1** and complex **2** at temperatures of 1.9-300 K.

T/K	H_T-$H_{298.15}$/(kJ·mol^{-1})		S_T-$S_{298.15}$/(kJ·mol^{-1})	
	Complex 1	Complex 2	Complex 1	Complex 2
1.936	-446.924	-453.906	-6712.51	-6365.49
4.936	-441.778	-449.340	-5091.89	-4927.16
7.936	-437.112	-445.174	-4349.84	-4264.62
10.936	-432.858	-441.349	-3893.88	-3854.61
13.936	-428.959	-437.814	-3578.16	-3568.41
16.936	-425.363	-434.525	-3344.06	-3354.31
19.936	-422.022	-431.441	-3162.25	-3186.46
22.936	-418.898	-428.527	-3016.11	-3050.18
25.936	-415.953	-425.753	-2895.36	-2936.43
28.936	-413.157	-423.092	-2793.27	-2839.28
31.936	-410.481	-420.521	-2705.24	-2754.68
34.936	-407.902	-418.019	-2628.03	-2679.77
37.936	-405.398	-415.568	-2559.26	-2612.46
40.936	-402.952	-413.155	-2497.17	-2551.21
43.936	-400.547	-410.764	-2440.46	-2494.85
46.936	-398.169	-408.386	-2388.09	-2442.48
49.936	-395.806	-406.011	-2339.29	-2393.41
52.936	-393.448	-403.629	-2293.43	-2347.09
55.936	-391.086	-401.235	-2250.03	-2303.09
58.936	-388.713	-398.821	-2208.70	-2261.05
61.936	-386.321	-396.383	-2169.11	-2220.70
64.936	-383.906	-393.916	-2131.03	-2181.81
67.936	-381.462	-391.417	-2094.23	-2144.18
70.936	-378.985	-388.882	-2058.55	-2107.67
73.936	-376.472	-386.309	-2023.85	-2072.14
76.936	-373.919	-383.695	-1990.01	-2037.48
79.936	-371.325	-381.038	-1956.93	-2003.60
82.936	-368.687	-378.337	-1924.54	-1970.43
85.936	-366.003	-375.590	-1892.75	-1937.89

88.936	-363.272	-372.796	-1861.51	-1905.92
91.936	-360.493	-369.953	-1830.78	-1874.49
94.936	-357.664	-367.061	-1800.50	-1843.54
97.936	-354.785	-364.120	-1770.64	-1813.03
100.936	-351.855	-361.127	-1741.17	-1782.93
103.936	-348.873	-358.083	-1712.06	-1753.21
106.936	-345.839	-354.988	-1683.28	-1723.85
109.936	-342.753	-351.840	-1654.82	-1694.82
112.936	-339.613	-348.640	-1626.64	-1666.10
115.936	-336.421	-345.387	-1598.74	-1637.67
118.936	-333.175	-342.080	-1571.11	-1609.51
121.936	-329.877	-338.721	-1543.71	-1581.61
124.936	-326.524	-335.307	-1516.55	-1553.96
127.936	-323.119	-331.840	-1489.62	-1526.54
130.936	-319.660	-328.319	-1462.90	-1499.33
133.936	-316.148	-324.745	-1436.38	-1472.34
136.936	-312.583	-321.116	-1410.05	-1445.55
139.936	-308.965	-317.434	-1383.91	-1418.94
142.936	-305.293	-313.697	-1357.95	-1392.53
145.936	-301.569	-309.907	-1332.17	-1366.28
148.936	-297.792	-306.063	-1306.55	-1340.21
151.936	-293.962	-302.166	-1281.09	-1314.30
154.936	-290.079	-298.214	-1255.78	-1288.54
157.936	-286.143	-294.209	-1230.62	-1262.94
160.936	-282.155	-290.149	-1205.60	-1237.48
163.936	-278.113	-286.036	-1180.72	-1212.15
166.936	-274.018	-281.869	-1155.97	-1186.96
169.936	-269.870	-277.648	-1131.34	-1161.90
172.936	-265.668	-273.372	-1106.83	-1136.96
175.936	-261.412	-269.041	-1082.43	-1112.13
178.936	-257.102	-264.656	-1058.13	-1087.41
181.936	-252.736	-260.214	-1033.94	-1062.80
184.936	-248.315	-255.717	-1009.83	-1038.28
187.936	-243.837	-251.162	-985.81	-1013.84
190.936	-239.301	-246.549	-961.87	-989.49
193.936	-234.706	-241.878	-937.99	-965.22
196.936	-230.052	-237.146	-914.18	-941.00
199.936	-225.336	-232.352	-890.41	-916.85
202.936	-220.557	-227.495	-866.69	-892.73
205.936	-215.713	-222.572	-842.99	-868.65
208.936	-210.802	-217.582	-819.32	-844.60

211.936	-205.822	-212.522	-795.65	-820.55
214.936	-200.770	-207.389	-771.98	-796.50
217.936	-195.643	-202.180	-748.29	-772.43
220.936	-190.437	-196.891	-724.57	-748.33
223.936	-185.150	-191.519	-700.80	-724.17
226.936	-179.777	-186.057	-676.96	-699.95
229.936	-174.313	-180.502	-653.04	-675.63
232.936	-168.753	-174.847	-629.02	-651.19
235.936	-163.092	-169.086	-604.87	-626.62
238.936	-157.322	-163.211	-580.57	-601.88
241.936	-151.437	-157.214	-556.09	-576.93
244.936	-145.429	-151.085	-531.41	-551.76
247.936	-139.288	-144.814	-506.49	-526.31
250.936	-133.003	-138.390	-481.30	-500.55
253.936	-126.564	-131.798	-455.79	-474.44
256.936	-119.956	-125.023	-429.92	-447.92
259.936	-113.166	-118.050	-403.64	-420.93
262.936	-106.176	-110.859	-376.91	-393.43
265.936	-98.968	-103.429	-349.65	-365.33
268.936	-91.520	-95.737	-321.80	-336.57
271.936	-83.810	-87.756	-293.29	-307.06
274.936	-75.809	-79.457	-264.03	-276.70
277.936	-67.489	-70.806	-233.93	-245.41
280.936	-58.815	-61.766	-202.89	-213.06
283.936	-49.749	-52.297	-170.79	-179.54
286.936	-40.249	-42.352	-137.51	-144.69
289.936	-30.268	-31.880	-102.91	-108.39
292.936	-19.753	-20.824	-66.83	-70.45
295.936	-8.643	-9.120	-29.10	-30.70
298.936	3.168	3.346	10.61	11.21
301.936	15.674	16.568	52.24	55.21

Standard uncertainties u are $u(p)$ =0.10 mPa, $u(T)$ = 0.01 K(1.9$<$K$<$20), $u(T)$ = 0.02 K(20$<$K$<$300)
$u(H_T\text{-}H_{298.15K}) = u_r(C_{p,m})(C_{p,m} / J\cdot K^{-1}\cdot mol^{-1})[T/K\text{-}298.15\ K]$
$u(S_T\text{-}S_{298.15K}/J\cdot K\cdot mol^{-1}) = u_r(C_{p,m})(C_{p,m} / J\cdot K^{-1}\cdot mol^{-1})[\ln(T/298.15\ K)]$

The infrared spectrograms of the complexes were measured at 4000-500 cm^{-1} using a BRUKER TENSOR27 Fourier transform infrared spectrometer. The Raman spectra of the complexes were measured using a VERTEX-70 FTIR-RAMAN II (BRUKER, Germany).
